# Surgical treatment, complications, reoperations, and healthcare costs among patients with clavicle fracture in England

**DOI:** 10.1186/s12891-022-05075-5

**Published:** 2022-02-09

**Authors:** Simone Wolf, Abhishek S. Chitnis, Anandan Manoranjith, Mollie Vanderkarr, Javier Quintana Plaza, Laura V. Gador, Chantal E. Holy, Charisse Sparks, Simon M. Lambert

**Affiliations:** 1 Synthes GmbH, Zuchwil, Switzerland; 2 Medical Devices Epidemiology, Johnson & Johnson, New Brunswick, NJ USA; 3 Mu Sigma, Bangalore, India; 4 DePuy Synthes, Inc., West Chester, PA USA; 5 Johnson and Johnson, Madrid, Spain; 6 Johnson and Johnson GmbH, Neuss, Germany; 7 University College London Hospital, London, UK

**Keywords:** Clavicle, Fractures, Bone, Cost of Illness, United Kingdom, Cohort Studies, Postoperative Complications, Reoperation

## Abstract

**Introduction:**

The clinical and economic burden of clavicle fractures in England is not well documented. This study evaluated rates of surgical treatment, post-surgical complications, reoperations and costs in patients with clavicle fractures using the Clinical Practice Research Datalink (CPRD) database.

**Methods:**

CPRD data were linked to National Health Service Hospital Episode Statistics data. Patients with a diagnosis of clavicle fracture between 2010–2018 were selected in CPRD (date of fracture = index date). Of those, patients with surgical intervention within 180 days from index fracture were identified. Rates of post-surgical complications (i.e., infection, non-union, and mal-union), reoperations (for device removal or for postoperative complications), post-operative costs and median time to reoperations were evaluated up to 2 years after surgery.

**Results:**

21,340 patients with clavicle fractures were identified (mean age 35.0 years(standard deviation (SD): 26.5), 66.7% male). Surgery was performed on 672 patients (3.2% of total cohort) at an average 17.1 (SD: 25.2) days post-fracture. Complications (i.e., infection, non-union, or malunion) affected 8.1% of surgically treated clavicle fracture patients; the rate of infection was 3.5% (95% CI, 1.7%- 5.2%), non-union 4.4% (95% CI, 2.4%-6.5%), and mal-union 0.3% (95% CI, 0%-0.7%). Adjusting for age, gender, comorbidities and time to surgery, the all-cause reoperation rate was 20.2% (13.2%-30.0%) and the adjusted rate of reoperation for implant removal was 17.0% (10.7%-25.9%)—84% of all-cause reoperations were thus performed for implant removal. Median time to implant removal was 254 days. The mean cost of reoperations for all causes was £5,000. The most expensive reoperations were for cases that involved infection (mean £6,156).

**Conclusions:**

Complication rates following surgical clavicle fracture care averaged 8.1%. However, reoperation rates exceed 20%, the vast majority of reoperations being performed for device removal. Technologies to alleviate secondary device removal surgeries would address a significant clinical unmet need.

## Background

Clavicle fractures are common and account for approximately 2.6% to 4.0% of all fractures. The incidence of clavicle fractures is estimated to be 64 per 100,000 persons per year [1, 2]. Clavicle fractures typically occur due to falls on the lateral aspect of the shoulder, falls on the outstretched hand, or high-energy direct impact over the bone. The peak incidence occurs in children and young adults; over one-third of clavicle fractures in males occur between the ages of 13 and 20 years, while 20% of clavicle fractures in females occur in this age group [3]. Most clavicle fractures occur in the middle portion, or shaft, of the bone.

Non-operative treatment options for clavicle fracture include pain reduction with analgesics and/or kinesiology tape, combined with temporary immobilization by sling or collar. Operative treatment of clavicle fractures may be accomplished with open reduction and internal fixation (ORIF) using plates and screws or intramedullary fixation (IMF) [4]. The location, fracture type and patient characteristics are key considerations for clavicle fracture management strategies [1, 3, 5]; however, the criteria for nonsurgical or surgical management are not clearly established [6]. Emergency care with a most likely surgical intervention is usually indicated in cases of open midshaft clavicle fractures, fractures with neurovascular compromise and/or tenting, as well as "floating shoulders" (i.e., ipsilateral clavicle and glenoid neck fractures) [7, 8]. Orthopedic referral is also indicated for significant fracture displacement, comminution, and shortening, the guidance on orthopedic referral being less directive for less severely displaced fractures [9–11].

Compared to nonsurgical care, surgical management of clavicle fractures has been shown in recent meta-analyses to be associated with better clinical and functional recovery and higher patient satisfaction, as well as lower rates of non-union and faster return to work [10–13]. Specifically, surgical treatment of clavicle fracture was associated with bony union in 96.7% cases [10, 11, 14, 15], compared to the approximately 15% non-union rate – and 0.4%-7.8% infection rate – observed in patients treated with nonoperative care [16–19]. While rare, surgical interventions do carry risks, which may increase with older age, alcohol consumption, diabetes, illicit drug use, previous surgery of the shoulder, and technical errors during surgery [20–22].

The clinical and economic burden of clavicle fractures is not well characterized. A prior analysis of US commercial insurance claim data evaluated 95,243 patients with clavicle fractures and found that 15.2% underwent surgical repair [23]. Among the patients undergoing surgery for clavicle fracture, 2-year rates of infection, non-union, and mal-union were low (1.0, 4.2, and 0.9%, respectively); however, the rate of reoperation was high due to device removal procedures [23]. The objective of the current study is therefore to evaluate rates of surgical treatment, reoperations, post-surgical complications (i.e., infection, non-union, and malunion), reoperations and costs of reoperations in patients with clavicle fractures requiring surgery in England.

## Methods

### Study design

A retrospective cohort study of patients with clavicle fracture requiring surgical repair with a longitudinal follow-up of up to two years post surgery was analysed to evaluate rates of complications and reoperations, along with healthcare costs.

### Data sources

Linked data from the UK Clinical Practice Research Datalink (CPRD) database and Hospital Episode Statistics (HES) Admitted Patient Care database from 2010 to 2018 were queried. The CPRD database collects data from general practitioners’ practices and includes demographic information, diagnoses, clinical measures (e.g., blood pressure), prescriptions, laboratory results, referrals to secondary care, and date of death [24]. The HES database receives administrative and clinical data from the National Health Service (NHS) and records hospital admission and discharge dates, demographic information, and international classification of disease (ICD)‐10 diagnoses. Patient‐level data in CPRD and HES are linked using a hierarchical stepwise linkage algorithm which includes NHS number, date of birth, sex, and postcode identifiers [25–27]. The study was approved by the Independent Scientific Advisory Committee (ISAC) – Protocol # 19–185. Informed Consent and Investigational Review Board (IRB) was not required for this study as it used data from an anonymous, de-identified, administrative database. Data from CPRD and HES being fully de-identified, this study was exempt from IRB approval.

### Patient population

Patients with a diagnosis of clavicle fracture and a clavicle surgical repair procedure between 2010–2018 were identified. The date of the fracture was defined as the index date. The proportion of patients receiving surgical treatment was evaluated. Surgical treatment was defined as a bone repair procedure of the clavicle within 180 days after fracture (aka index) with or without internal fixation devices. Patients were required to have medical records available for a minimum of 180 days pre-index (baseline period). Patients were excluded if they had any evidence of polytrauma or diagnoses of non-union, malunion, osteomyelitis, or infection during the baseline period and up to 30 days post-index. Only patients with “research-grade” records, as defined by CPRD, were included in the study.

### Baseline demographic and clinical characteristics

Patient demographics that were evaluated included age, sex, smoking status, and year of surgery. Baseline comorbidity was assessed using the Charlson Comorbidity Index (CCI) and all diseases listed in the CCI [28]. Time from index to surgery was also assessed.

### Healthcare resource utilization

Rates of infection, non-union, and malunion following surgery, as well as rates of reoperations were analysed at 1- and 2-years post-surgery. Reoperations were defined as device removal reoperations versus complication-related reoperation (e.g., due to infection or non-union). Device removal reoperations were defined as surgeries with specific device-removal codes, with no concurrent diagnoses of infection, non-union or malunion.

### Healthcare costs

Mean all-cause total healthcare costs from day of surgery to 2-year post-surgery were calculated for all patients. Costs were expressed in UK pounds. Healthcare costs were obtained from the Personal Social Services Research Unit (PSSRU) 2018 Cost of Care public document and Healthcare Resource Group (HRG) codes available in HES and NHS 2018 reference costs. Drug costs were obtained from the 2018 British National Formulary.

### Statistical analysis

All study variables were analysed descriptively. Counts and proportions (dichotomous variables) and means and standard deviations (continuous variables) were provided. Poisson regression models were built to evaluate risk of reoperations, adjusted for age, gender and comorbidity. Generalized linear models with log link and gamma distribution were used to evaluate the cost of care associated with infection, non-union, complication-related reoperations and device-removal reoperations.

## Results

### Patient selection and rates of surgical treatment

A total of 21,340 patients with clavicle fractures were identified from 2010 to 2018 (mean [standard deviation (SD)] age 34.6 [26.9] years, and 66.7% male). Among these 21,340 patients with clavicle fracture, 672 underwent surgical fixation at an average 17.1 day after index. The percentage of patients with clavicle fracture undergoing surgery decreased as follows: from 2010 to 2012, 8,575 fractures were identified, of which 301 underwent surgery (3.51%); from 2013 to 2015, 7,697 fractures with 250 surgeries were identified (3.25%) and from 2016 to 2018, the count of fractures reached 5,068 of which 121 were treated surgically (2.39%).

### Baseline demographic and clinical characteristics

Table 1 shows demographic and clinical characteristics of patients with clavicle fractures and clavicle repair surgery, from 2010 to 2018. Most patients were less than 45 years old and had no comorbidities at index. Only 33 of the 672 (5%) patients had any comorbidities, the most common being chronic pulmonary disease, which includes asthma and other common respiratory conditions such as chronic bronchitis and emphysema. Diabetes (type I or II) affected 8 patients (1.1%), but only 1 patient had diabetes with complications.

**Table 1:** Demographic and clinical characteristics of patients with surgical treatment for clavicle fracture. (*IQR* = Interquartile range)

*N*	672	
**Age, mean (SD)**	38.4 (16.5)	
**Age category, *n* (%)**		
Less than 18	59	8.8%
18–24	118	17.6%
25–34	114	17.0%
35–44	132	20.0%
45 and greater	249	37.0%
**Female, *n* (%)**	126	18.8%
**Smoking Status**		
Current smoker	67	10.0%
Past smoker	47	7.0%
**Charlson Comorbidity Index, mean (SD)**	0 (0.3)	
**Charlson Comorbidity Index Categories, *n* (%)**		
0	639	95.1%
1–2	31	4.6%
3–4	2	0.3%
5 +	0	0.0%
**Comorbidities**		
Peripheral Vascular Disease	1	0.1%
Cerebrovascular Disease	3	0.4%
Dementia	3	0.4%
Chronic Pulmonary Disease	18	2.7%
Diabetes without complications	7	1%
Diabetes with complications	1	0.1%
Renal Disease	3	0.4%
Cancer	2	0.3%
**Days from Clavicle Fracture to Surgery**	17.1 (25.2)	
**Average Number of Follow-up Days Post Fracture (Mean (median, [IQR])**	593 (730 (469–730))	
**Follow-up**		
Patients with complete 12 months follow-up	534	79.5%
Patients with complete 24 months follow-up	405	60.3%

### Post-operative complication and reoperation rates

Complication and reoperation rates, for the entire cohort and by 3-year time frames, are shown in Table 2 below. Overall, infection and non-union affected less than 5% of patients. However, 22.8% underwent reoperation. Reoperation rates for patients operated between 2016–2018 were lower, but only 61 patients (out of 121) had complete 2-year follow-up.

**Table 2:** Crude 2-Year Complication and Reoperation Rates following Surgery for Clavicle Repair

	**All Patients**	**2010–2012**	**2013–2015**	**2016–2018**^**a**^
Infection	3.1% (95%CI:2.9%-3.4%)	3.6% (95%CI:3.1%-4.0%)	3.3% (95%CI:2.8%-3.9%)	3.3% (95%CI:2.5%-4.1%)
Non-Union	4.0% (95%CI:3.7%-4.3%)	4.0% (95%CI:3.5%-4.5%)	5.8% (95%CI:4.9%-6.8%)	3.3% (95%CI:2.5%-4.1%)
Malunion	0.9% (95%CI:0.8%-1.0%)	0.4% (95%CI:0.4%-0.5%)	0.0%	0.0%
All Reoperations	22.8% (95%CI:21.4%-24.1%)	24.1% (95%CI:21.7%-26.5%)	24.2% (95%CI:20.9%-27.4%)	14.8% (95%CI:11.6%-17.9%)

^a^Only 61 patients out of 121 with complete 2-year follow-up

**Fig. 1:**
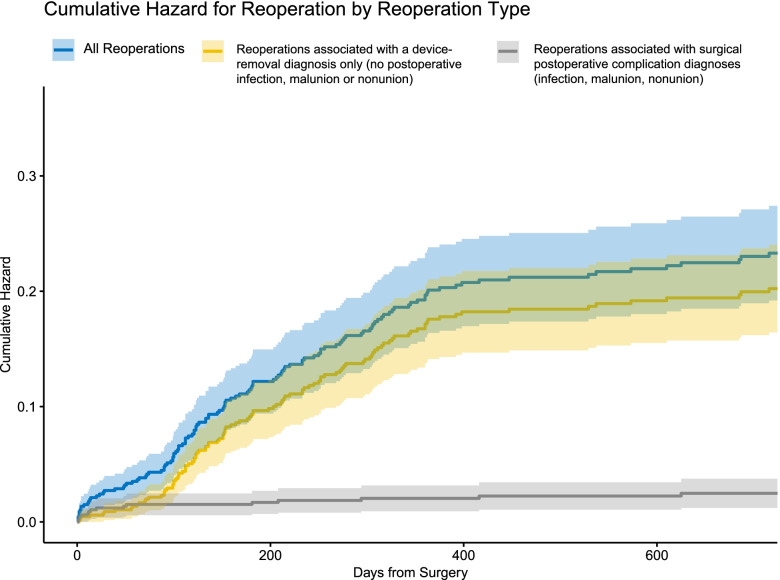
Cumulative hazard for reoperation for patients with all reoperation types, reoperations due to device removal or reoperation due to postoperative complications. More than 84% of all reoperations were conducted for device removal (yellow lines)

Adjusting for age, gender, comorbidities and time to surgery, the adjusted all-cause reoperation rate was 20.2% (13.2-30.0%) and the adjusted rate of implant removal was 17.0% (10.7-25.9%). Implant removal was therefore the main diagnosis associated with reoperation, as shown on Fig. 1: the cumulative hazard for reoperation, reoperation associated with device removal only vs reoperation associated with complications are shown over the 2 year time frame. Using adjusted rates, reoperations associated with complications represent less than 16 percent of all reoperations. Median time to implant removal was 254 days.

### Healthcare costs

The cost to the NHS of reoperations due to complications included inpatient, outpatient, and prescription costs. The mean cost of reoperations for all causes was £5,000. The most expensive reoperations were for cases that involved infection (mean £6,156). Table 3 presents the costs of reoperations due to complications presented by type of complication.

**Table 3:** Two-year NHS cost of surgical treatment of clavicle fracture (*n* = 672)

Cost category	Mean cost (95% CI)
Infections with reoperations	£6,156 (£937-£24,810)
Infections (with or without reoperations)	£3,750 (£709-£12,188)
Non-unions (with reoperations)	£4,407 (£1,094-£13,106)
Reoperations, all causes	£5,017 (£1,132-£15,979)
Reoperations for device removal	£3,151 (£1,746-£5,524)

### Factors associated with complications and reoperations

**Fig. 2:**
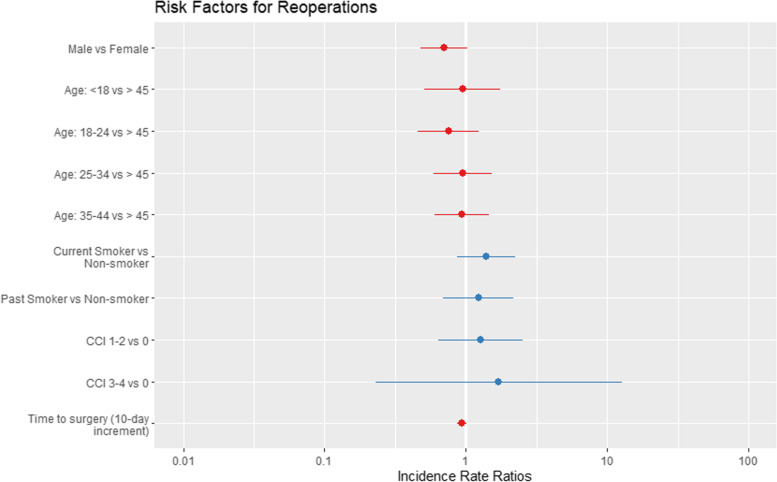
Risk ratios for reoperations. Models for reoperations did not identify patient or clinical variables associated with risks of reoperations. CCI = Charlson Comorbidity Index

Poisson regression models did not identify any significant patient variables predictive of reoperation. Figure 2 presents the incidence risk ratios for all-cause reoperation and demographic and clinical variables (i.e., age, gender, and comorbidity).

## Discussion

Relevant, high-quality evidence for the treatment patterns, complications, resource utilization, and costs associated with clavicle fracture fixation is scarce [15]. Real-world databases leverage data originating from clinical practice and provide an opportunity to assess the effectiveness of surgical treatments in large numbers of patients treated in real-world settings [29, 30]. This observational evidence can assist clinicians, purchasers, consumers, and policymakers in making more informed decisions that can improve healthcare at the individual and population levels [31]. Our intent with the current study was to conduct such an analysis in patients in England using the CPRD database.

A prior real-world US administrative claims database analysis conducted by our research group [23] found that the rate of clavicle fracture fixation was higher among US patients with commercial insurance compared to that observed in this current study (15.2 vs. 3.2%), albeit these two studies include very different populations in different geographies. In regard to complications associated with clavicle fracture fixation, this study compares as following to the US study: the rate of infection was lower in the US study (1.0 vs. 3.5%), the rate of non-union was similar (4.2 vs. 4.4%), and the rate of malunion was higher (0.9 vs. 0.3%). Importantly however, the comorbidities and overall characteristics of patients in both studies were different, as explained below, and therefore different risk factors or confounders may explain these differences.

The discrepancies in rates of fixation and reoperation between the current study of patients in England and the US study of patients with commercial insurance are possibly due to differences in treatment patterns between the countries, and possibly provider payment practices. The reoperation rate within 2 years observed in this study is similar to published Canadian studies, [32, 33] whereas the rate of reoperation we observed in the US commercial claims analysis [23] is consistent with other published US studies [34, 35]. Differences in complications between the current study and the US commercial claims study, however, may be partly attributed to differences in data collection as the US study was based on administrative claims data, whereas the CPRD database contains electronic health data (EHR) data from general practitioners’ practices. EHRs can provide a more thorough understanding of patient outcomes as they capture a variety of patient-level data that represent integral components of provider care that are not available in administrative claims databases [36]. Another factor contributing to the differences between the studies is the different patient populations evaluated. The CPRD database contains information for all residents in the UK, whereas the US study only included data for individuals with commercial health insurance. The patients in the US study were younger (mean age 23.8 years vs. 38 years in the current study), a smaller proportion were male (70.8 vs 81.2% in the current study), and a smaller proportion had a CCI score of 0 (86.4 vs. 95.1% in the current study).

Regardless of the specific reoperation or removal rate, complications did occur, but the reoperations due to actual complications was far lower than associated solely with the device removal diagnoses. These findings are consistent with a prior systematic review that summarized the published evidence for complications with clavicle fracture fixation. The authors hypothesized that plate type, thickness and pre-contouring to the anatomic shape of the clavicle would also have an influence [15]. The impact of pre-contouring of fixation plates as a strategy to reduce reoperations for device removal has also been evaluated by other researchers and found to be effective [15, 20, 37]. Advancements in hardware and supporting technologies could further help address the high rate of secondary surgery performed to remove hardware.

This study has some limitations that are expected due to the data sources and research techniques used. The exact plates and devices used are not available. From 2010 to 2018, the most commonly used plates were: reconstruction plates, with ease of contouring but prone to fatigue failure when used incorrectly; anterosuperior plates, with ease of use and relatively poor fit but less fatigue failure, or locking compression plates (LCP) plates, difficult to contour. From the databases, unfortunately, the device used for each patient is unknown. In addition, detailed risk factors, such as clear definitions of smoking status (e.g., heavy vs light) are unknown. Challenges associated with the use of EHR data include missing data, erroneous inputs, uninterpretable data, inconsistencies among providers and over time, and deciphering data stored in non-coded text notes [38]. There may be factors that contributed to the findings that were not discernable from the CPRD and HES data. Hence, we were not able to account for unmeasured, inadequately measured, and unmeasurable residual confounding. Finally, the study results of this study are only generalizable to patients in the UK meeting the inclusion and exclusion criteria.

## Conclusions

Clavicle surgery in patients with fracture repair has low rates of complications such as infection, malunion or non-union. However, reoperations are frequent because of device removal procedures, which account for more than 85% of all reoperations. New implants that may not require removal may be associated with lower reoperation rates.

## Data Availability

The data for these analyses were made available to the authors by third-party licenses from CPRD (https://www.cprd.com/Data). Under the licensing agreement, the authors cannot provide raw data to the Journal. Other researchers could access the data by purchase through CPRD, and the inclusion criteria specified in the Methods section would allow them to identify the same cohort of patients we used for these analyses.

## Abbreviations

ICD: International Classification of Disease
CCI: Charlson Comorbidity Index
CPRD: Clinical Practice Research Datalink
EHR: Electronic Health Records
HES: Hospital Episode Statistics
IMF: Intramedullary fixation
IRB: Investigational Review Board
NHS: National Health Service
ORIF: Open reduction and internal fixation
SD: Standard Deviation
TEN: Titanium elastic nail
